# Correlation of PD-L1 and SOCS3 Co-expression with the Prognosis of Hepatocellular Carcinoma Patients

**DOI:** 10.7150/jca.46158

**Published:** 2020-07-11

**Authors:** Liuxi Chen, Xingxing Huang, Wenzheng Zhang, Ying Liu, Bi Chen, Yu Xiang, Ruonan Zhang, Mingming Zhang, Jiao Feng, Shuiping Liu, Ting Duan, Xiaying Chen, Wengang Wang, Ting Pan, Lili Yan, Ting Jin, Guohua Li, Yongqiang Li, Tian Xie, Xinbing Sui

**Affiliations:** 1 Department of Medical Oncology, the Affiliated Hospital of Hangzhou Normal University, College of Medicine, Hangzhou Normal University, Hangzhou, Zhejiang, China.; 2 Key Laboratory of Elemene Class Anti-cancer Chinese Medicine of Zhejiang Province, Hangzhou Normal University, Hangzhou, Zhejiang, China.; 3 State Key Laboratory of Quality Research in Chinese Medicines, Faculty of Chinese Medicine, Macau University of Science and Technology, Macau, P.R. China.

**Keywords:** PD-L1, SOCS3, CD276, CD4, CD8, hepatocellular carcinoma, prognostic value

## Abstract

**Purpose:** To investigate the correlation between the expression of PD-L1, SOCS3 and immune-related biomarkers CD276, CD4, CD8 in hepatocellular carcinoma (HCC) and further determine the relationship with clinicopathologic characteristics and the prognostic value of their co-expression in HCC patients.

**Methods:** We assessed the expression of PD-L1, CD276, SOCS3, CD4 and CD8 by immunohistochemistry in tumor tissue from 74 HCC patients who underwent curative hepatectomy.

**Results:** High expression of PD-L1 was significantly associated with high Edmondson grade (p<0.01) and elevated enzyme (p=0.037); high expression of CD276 was significantly correlated with high Edmondson grade (p=0.021); high expression of SOCS3 was significantly associated with age (p=0.026) and tumor size (p=0.041), while PD-L1 showed no significant correlation. The expression of PD-L1, CD276, SOCS3 protein and other clinicopathological factors (sex, vascular invasion, tumor number, tumor capsule, pT stage, liver cirrhosis, HBsAg, TBiL, AFP) showed no significant correlation (p>0.05). High expression of CD8 was respectively significantly associated with worse overall survival (OS) (p=0.002). There was no significantly difference between CD4 and CD8 high-expression and overall survival (OS) (p=0.100). Both high expression of PD-L1 (p=0.003) and low expression of SOCS3 (p=0.015) was significantly associated with worse overall survival (OS). But CD276 only had a trendency (p=0.166). Additionally, multivariate Cox regression models implied that PD-L1, SOCS3, as well as both CD4 and CD8 was an independent prognostic factor for OS (p<0.05). Furthermore, HCC patients with PD-L1 low-expression and SOCS3 high-expression had a better prognostic according to the different pT stages (p<0.05).

**Conclusions:** We for the first time demonstrated that PD-L1 and SOCS3 were independent prognostic factor for HCC patients. Co-expression of low PD-L1 and high SOCS3 could be a better predictive marker for HCC patients.

## Introduction

Hepatocellular carcinoma (HCC) remains one of the most common and aggressive malignancies worldwide 1. It is reported that hepatocellular carcinoma has the fifth highest mortality rate in males and the sixth highest in females, due to the lack of effective diagnostic options and the late treatment 1, 2. Incidence rates showed higher numbers in China mainly associated with chronic hepatitis B virus (HBV) infection 3, 4. Recurrence, metastasis and frequent drug resistance are the main dilemma to the integrative cancer treatment of HCC 5. Recently, immunotherapy has been attempted to further improve prognosis with advanced HCC 6. By analyzing inflammatory gene including expression profiles, infiltrates, and regulatory molecules, an 'immune class' accounting for 30% of HCCs was characterized by immune cell infiltration, programmed cell death protein 1 (PD-1) and/or programmed cell death 1 ligand 1 (PD-L1) expression, IFN-γ signaling activation and markers of cytolytic activity 7.

Programmed cell death protein 1 (PD-1) is a receptor mainly expressed by T cells, which negatively regulates the effector phase of T cell responses. To date, monoclonal antibodies to either PD-L1 (atezolizumab, avelumab, and durvalumab) or PD-1 (nivolumab and pembrolizumab) are approved for the cancer treatment 8. PD-L1, as an independent predictive biomarker, is demonstrated low prediction accuracy attributed to the technical obstacles (variable detection antibodies, differing IHC cutoffs, staining of tumor versus immune cells) and the complexity of biological mechanisms (unevenly distribution, unstable expression) 9. Therefore, we tried to explore potential biomarkers in combination to verify what prognostic clinical utility they may hold.

The PD-1/PD-L1 interactions suppress CD8 cytotoxic T lymphocyte (CTL) survival, proliferation and effector function and can also induce the apoptosis of tumor-infiltrating T cells 10. PD-1/PD-L1 interactions lead the differentiation of CD4 T cells to Foxp3 regulatory T cells (Tregs) 11, which are known to further inhibit the immune system and promote peripheral immune tolerance 12. Tumor-infiltrating lymphocytes (TILs) are a group of lymphocytes located around tumor cells. The presence of TILs is associated with tumor regression and prognosis in HCC, which is often regarded as an "immunogenic tumor" 13.

B7-H3 (also known as CD276), a co-stimulator molecule of the cell surface B7 protein superfamily, is mainly expressed on dendritic cells along with T lymphocytes, B monocytes and NK cells 14. It was reported that CD276 had a co-stimulating effect on the proliferation of both CD4 and CD8 T cells. Combinatorial blockade of anti-CD276 and anti-PD-1 has approved that this approach can be mutually enhancing treatment effect of established tumors 15. Kang et al. have shown that down regulation of CD276 can significantly reduce cell migration and invasion in HCC, which may be referred to epithelial-to-mesenchymal transition through the JAK2/STAT3/Slug pathway 16.

Suppressor of cytokine signaling 3 (SOCS3) is a tumor suppressor gene and its inactivation may play a role in HCC initiation and development 17. The deletion of the SOCS3 is an important mechanism for the activation of the STAT3 and this mechanism vitally act in the occurrence and progression of hepatitis-related liver cancer 18. Zhang et al. found that overexpression of SOCS3 strongly inhibited STAT1 phosphorylation and PD-L1 up-regulation 19.

To our knowledge, PD-L1 is respectively related to CD4, CD8, CD276, SOCS3, but the relationship between those co-expression and hepatocellular carcinoma has not been reported. Hence, we retrospectively selected 74 hepatocellular carcinoma cases and performed immunohistochemical analysis for PD-L1, CD4, CD8, CD276 and SOCS3 for each paraffin-embedded block. In addition, we also investigated the correlation of the expression of CD4, CD8, PD-L1, CD276 and SOCS3 with clinicopathological features and clinical outcomes. We are committed to find potential biomarkers in combination with PD-L1 to predict hepatocellular carcinoma progression and prognosis.

## Materials and Methods

### Public Gene Expression Omnibus dataset analysis

Two publicly available Gene Expression Omnibus (GEO) datasets (GSE102079 and GSE121248) containing gene expression information from paracancerous tissues and cancerous tissues were downloaded from Gene Expression Omnibus (http://www.ncbi.nlm.nih.gov/geo/). The processed data dealed with normalization procedures were directly obtained from the corresponding websites, and no other transformations were performed.

### Patient population and tumor samples

All patient information and clinical data were obtained from National Human Genetic Resources Sharing Service Platform. A total of 74 patients with formalin-fixed, paraffin-embedded tumors (FFPE) were selected retrospectively, who had undergone surgery without neoadjuvant treatment between June 2007 and March 2008. Patients were histopathologically diagnosed as hepatocellular carcinoma. Tumor staging and grading was carried out according to the American Joint Committee on Cancer (AJCC), TNM Staging System for Urethral Carcinoma (8th ed., 2017). When the patient died, the cause of death was determined by the attending physician or by death certificate.

### Immunohistochemistry

PD-L1, CD4, CD8, CD276 and SOCS3 immunohistochemistry (IHC) were available in 74 cases. Each case was performed PD-L1, CD4, CD8, CD276 and SOCS3 immunohistochemical staining using the same paraffin-embedded blocks. The sections were incubated with PD-L1 antibody (1:200 dilution; 13684S, Cell Signaling, USA), CD4 antibody (RMA-0620, Maixin Biotechnologies, China), CD8 antibody (MAB-0021, Maixin Biotechnologies, China), CD276 (1:400 dilution; HPA009285, Sigma, USA) and SOCS3 (1:500 dilution; ab16030, Abcam, UK) respectively overnight at 4 °C. A DAB Kit (K0675, Dako, Denmark) was applied to the sections, followed by haematoxylin counterstain.

Comparing cancer tissue with paracancerous tissue, the staining conditions were established for each antibody, which were used as internal control in each staining protocol. In CD4/CD8 and CD276 IHC, cell membrane and cytoplasmic expression with higher intensity than internal positive control was regarded as positive. Cell membrane and cytoplasmic staining in 74 cases with higher intensity than internal positive control was interpreted as positive in PD-L1 or SOCS3 IHC. In each case, at least 500 cells were evaluated in 5 representative high power fields (200×), and the proportion of positively stained cells were calculated.

According to the staining protocol, the staining conditions for each antibody were established and paracancerous tissue was used as internal control. Based on a four-point system, cytoplasmic/membranous protein expression was scored by the staining intensity (0: negative; 1: weak; 2: moderate; 3: strong) and density (0: none; 1: less than 40%; 2: 40-70%; 3: more than 70%). Quantitation of immunohistochemical staining was obtained by multiplying staining percentage by staining intensity, 0-3 were defined as low-expression whereas 4-9 were defined as high-expression. All immunostaining scores were obtained from five randomized fields by two investigators who were blinded to the clinical data.

### Statistical analyses

Statistical analyses were conducted by SPSS software (version 23.0; IBM, Armonk, NY). Either Chi-square test or Fisher's exact test was used to explore the correlation between immunostaining markers and clinicopathological variables by Pearson's contingency analysis. The univariate Cox regression model was performed on the clinicopathological features and immunostaining markers as covariates. Multivariate Cox proportional-hazards analysis was conducted including the significant covariates (p value <0.11) according to the univariate analysis. Overall survival (OS) was from the date of curative hepatectomy to death. Kaplan-Meier analysis was used the log-rank test to compare differences. Two-tailed p value <0.05 was considered as statistically significant in all cases.

## Results

### Expression of PD-L1, CD4, CD8, CD276 and SOCS3 in Hepatocellular Carcinoma

**Figure 1:**
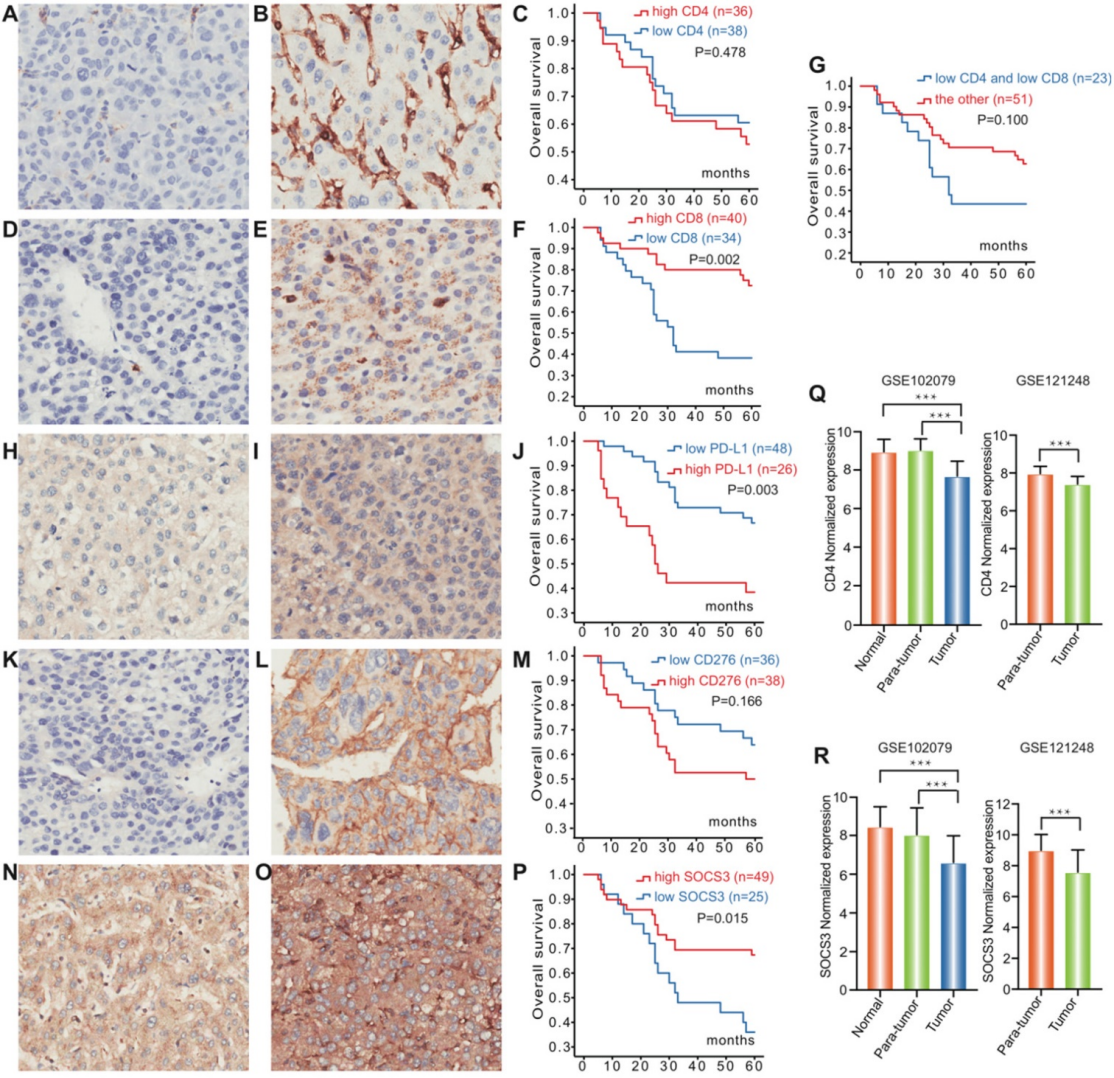
** Expression of PD-L1, CD4, CD8, CD276 and SOCS3 in hepatocellular carcinoma.** Representative immunohistochemical staining and Kaplan-Meier survival curves for CD4, CD8, PD-L1, CD276 and SOCS3 in hepatocellular Carcinoma. (**A**) CD4 low-expression; (**B**) CD4 high-expression; (**C**) No significant difference between the overall survival rate for patients with CD4 high-expression and that for patients with CD4 low-expression (p=0.478); (**D**) CD8 low-expression; (**E**) CD8 high- expression; (**F**) The overall survival rate for patients with CD8 high-expression was significantly higher than that for patients with CD8 low-expression (p=0.002); (**G**) No significant difference between the overall survival rate for patients with CD4/CD8 low-expression and that for patients with the other (p=0.100); (**H**) PD-L1 low-expression; (**I**) PD-L1 high-expression; (**J**) The overall survival rate for patients with PD-L1 low-expression was significantly higher than that for patients with PD-L1 high-expression (p=0.003); (**K**) CD276 low-expression; (**L**) CD276 high-expression; (**M**) No significant difference between the overall survival rate for patients with CD276 high-expression and that for patients with CD276 low-expression (p=0.166); (**N**) SOCS3 low-expression; (**O**) SOCS3 high-expression; (**P**) The overall survival rate for patients with SOCS3 high-expression was significantly higher than that for patients with SOCS3 low-expression (p=0.015). CD4 and SOCS3 expression statuses in hepatocellular carcinoma tissues and paracancerous tissues were obtained from GSE102079 and GSE121248. (**Q**) CD4 expression levels of cancerous tissues were significantly downregulated compared with the paracancerous and normal tissues (P<0.001); (**R**) SOCS3 expression levels of cancerous tissues were significantly downregulated compared with the paracancerous and normal tissues (P<0.001).

To investigate the role of PD-L1, CD4, CD8, CD276 and SOCS3 in Hepatocellular Carcinoma, we processed GSE102079 and GSE121248 datasets from Gene Expression Omnibus (GEO). Both GSE102079 and GSE121248 datasets indicated that CD4 and SOCS3 expression levels of cancerous tissues were significantly downregulated compared with the paracancerous tissues and normal tissues (P<0.001, Figure 1 Q, R).

IHC was carried out in 74 specimens to examine the expression of PD-L1, CD4, CD8, CD276 and SOCS3 in HCC patients who underwent surgical treatment, which was selected regarding to the AJCC guideline (Table 1). In the HCC tissues, PD-L1 was mainly expressed in the cellular membrane and cytoplasm of tumor cells in a diffuse manner; CD4/CD8, CD276 and SOCS3 protein were also mainly found in the cytoplasm and membrane of tumor cells (Figure 1). Twenty-six (35.1%) of 74 tumors in hepatocellular carcinoma had elevated levels of PD-L1 protein as detected by IHC, whereas high PD-L1 expression significantly associated with low alanine transaminase (ALT) levels (p=0.037) and high Edmondson grading (P<0.001) (Table 2). However, no statistically significant correlation was observed between the expression of PD-L1 and other clinicopathological features. CD4 and CD8 exhibited high expression rates in the HCC tissue with values of 48.6% (36/74) and 54.1% (40/74), indicating that there were no significant association between the expression of CD4 and CD8 and other clinicopathological features (Table 1). Furthermore, CD276 high expression and SOCS3 low expression in HCC tissue were significantly associated with high Edmondson grading (P=0.021) and age, tumor size (P=0.026, 0.041), respectively (Table 2).

### Survival Analysis

Kaplan-Meier survival and disease analysis of 74 HCC patients (Figure 1) showed significantly worse OS for the PD-L1 high-expression group compared with low-expression group (P=0.003). CD8 were significantly associated with survival from hepatocellular carcinoma (p=0.002), while low-expression of CD4/CD8 just showed a trend towards patient survival (p=0.100). Furthermore, the SOCS3 low-expression group had shorter OS than the corresponding high-expression group (P=0.015). However, no statistically significant association was observed between the expression of CD276 and OS. The followed-up period was 60 months or more, and 32 patients were dead at the time of analysis.

**Figure 2:**
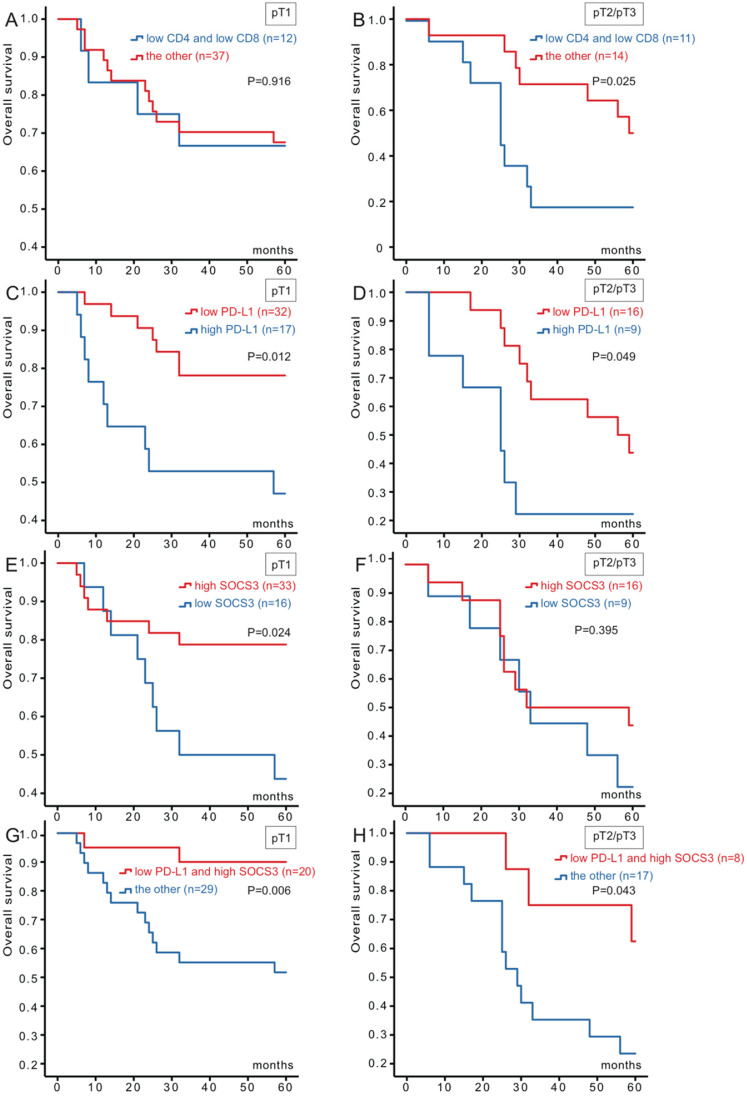
** Kaplan-Meier survival curves of 74 hepatocellular carcinoma patients in different pT stages according to CD4/CD8, PD-L1 and SOCS3 expression.** (**A**) No significant difference between the overall survival rate for patients with CD4/CD8 low-expression and that for patients with the other in pT1 stage (p=0.916); (**B**) The overall survival rate for patients with CD4/CD8 low-expression was significantly lower than that for patients with the other in pT2/pT3 stage (p=0.025); (**C**) The overall survival rate for patients with PD-L1 low-expression was significantly higher than that for patients with PD-L1 high-expression in pT1 stage (p=0.012); (**D**) The overall survival rate for patients with PD-L1 low-expression was significantly higher than that for patients with PD-L1 high-expression in pT2/pT3 stage (p=0.049); (**E**) The overall survival rate for patients with SOCS3 high-expression was significantly higher than that for patients with SOCS3 low-expression in pT1 stage (p=0.024); (**F**) No significant difference between the overall survival rate for patients with SOCS3 high-expression and that for patients with SOCS3 low-expression in pT2/pT3 stage (p=0.395); (**G**) The overall survival rate for patients with PD-L1 low-expression/SOCS3 high-expression was significantly higher than that for patients with the other in pT1 stage (p=0.006); (**H**) The overall survival rate for patients with PD-L1 low-expression/SOCS3 high-expression was significantly higher than that for patients with the other in pT2/pT3 stage (p=0.043).

Based on these results, we divided the cohort into two groups (49 for pT1 and 25 for pT2/pT3) for further Kaplan-Meier analysis (Figure 2). Combination CD4 low-expression with CD8 low-expression versus the others were significantly association with survival for pT2/pT3 (P=0.025), whereas no statistically significant association was observed for pT1. Patients in PD-L1 low-expression and SOCS3 high-expression both had better OS (P=0.012, P=0.024) than the other group for pT1 group. And for pT2/pT3 group, PD-L1 were also associated with hepatocellular carcinoma survival (P=0.049). However, OS about the SOCS3 high-expression group had no significant association with the corresponding low-expression group for pT2/pT3.

According to the results, we combined PD-L1 with SOCS3, and stratified as four types (both high-expression, both low-expression, high-/low-expression, low-/high-expression). Patients could be divided into low- and high-risk for death. Of the 49 patients, 20 of them were PD-L1 low-expression and SOCS3 high-expression status, while the rest with the other three groups. When analyzed as categoric variables in Kaplan-Meier analysis, the hepatocellular carcinoma had a better prognosis (P=0.006). There was a similar relationship for pT2/pT3 group between PD-L1 and SOCS3 staining (P= 0.043) (Figure 2).

### Cox Analysis

Given the significant correlation between the expression of CD4/CD8, PD-L1, CD276 and SOCS3, we performed univariate and multivariate Cox proportional-hazards analyses. Univariate analysis (Table 3) revealed that Edmondson grading (P=0.005), tumor number (P=0.004), pT categories (P=0.032), PD-L1 expression (P=0.004) and SOCS3 expression (P=0.019) were independent indicators for overall survival. We also confirmed that the tumor size (P=0.096), tumor capsule (P=0.083) and CD4/CD8 expression (P=0.107) have the trend with HCC survival. According to the data above, multivariate Cox regression model (Table 4), including the covariates of pT categories (pT1 vs. PT2/pT3), Edmondson grading (I-II vs. III-IV), tumor capsule (complete vs. incomplete), co-overexpression of both CD4 and CD8, PD-L1 expression and SOCS3 expression, exhibited that pT categories (P=0.045), CD4/CD8 expression (P=0.040), PD-L1 expression (P<0.001) and SOCS3 expression (P<0.001) were independent predictive factors for OS.

## Discussion

Hepatocellular carcinoma is prone to recurrence; metastasis and drug resistance may be the main obstacles in clinical management 5. Immunotherapy is attractive for hepatocellular carcinoma, which is considered as an “immunogenic tumor” 7. Moreover, tumor-infiltrating lymphocytes are crucial components of immune cell in tumor microenvironment which participate in tumorigenesis, tumor progression and play the key role in anti-tumor immune therapy 20. PD‐1 is defined as a negative molecular of the immune response and primarily expressed on activated T and B cells 21. PD-1/PD-L1 pathway is an important mechanism of immunosuppression in TME 21. CD276, as a costimulatory molecule, is participate in the secretion of IFN-γ during the T cell activation, and also regulates tumor immune microenvironment involving the aggressiveness of various tumors 22-24. The down-regulation of CD276 may be related to the JAK/STAT3 pathway in HCC 16. And the JAK/STAT3 pathway is the canonical target of SOCS3 expression, which is regarded as a tumor suppressor gene 18. The activation of STAT3 in hepatocytes is mediated via deletion of the SOCS3, resulting in hepatocarcinogenesis 18.

In the present study, we demonstrated that early-stage HCC patients with down regulation of CD8 in tumor tissue had a significantly higher risk of death, whereas no significant difference was observed in CD4 expression. These results further support that CD8 T lymphocytes play a vital role in antitumor immune response 25.

Given the complexity of the interaction between CD4 T lymphocytes and target antigens, depletion of CD4 T lymphocytes could decrease antitumor activity 26. We also verified that co-overexpression of CD4/CD8 significantly improved the prognosis of HCC patients, and COX analysis further supported it as an independent prognostic factor for OS 26, 27.

Patients with high PD‐L1 expression or low SOCS3 expression had significantly poorer prognosis, which were suggested as independent prognostic factors. Further study had shown that PD-L1 IHC had a good prognostic value in different stages of HCC patients, while SOCS3 only associated with stage I patients, and had no significant impact in stage II disease. The PD-L1 expression alone does not sufficiently predict for response in HCC patients 28. Therefore, a series of clinical trials has shown that PD-1/PD-L1 antibodies have promising outcomes for cancer treatment, only a part of patients responded to the treatments. It is noteworthy that only the combination of different markers can properly predict overall survival in HCC patients. We first found a significant correlation between the expression of PD-L1 and SOCS3 in HCC tumor tissue. High SOCS3 and low PD-L1 expressions had a better prognosis compared with the other. Additionally, the co-expression of SOCS3 and PD-L1 in HCC tissue had a best prognostic value in different pT stages compared with the individual markers. Recently, a series of studies have demonstrated that SOCS3 can also participate in the immune response by inhibiting the expression of inflammation-related cytokines 19, 29, 30. It is striking that overexpression of SOCS3 strongly inhibited STAT1 phosphorylation and PD-L1 up-regulation, and further studies confirmed that SOCS3 regulates JAK/STAT3 pathway through immunoregulation 19, 31. As our results suggested that PD-L1 and SOCS3 are negatively correlated in HCC tissues, and it still remains ambiguous whether SOCS3 can selectively down-regulate PD-L1 to promote anti-cancer immune responses in HCC. Relevant experiments need to be designed to verify our speculation for HCC patients in the future. However, no significant correlation was found between the expression of CD276 and HCC tumor tissues in this study. The main reason is that CD276 has a dual role in tumor cells by interacting with T-suppressor cells or T-effector cells 32. Future research needs to address the conundrum of CD276 and the sequence of its role in HCC.

In conclusion, we first reported the negative correlation between the expression of PD-L1 and SOCS3 in HCC tissue. At the same time, PD-L1 and SOCS3 were found to be independent prognostic factors for OS, and patients with PD-L1 low-expression and SOCS3 high-expression had the best prognosis compared with the individual markers in different pT stages. Therefore, frequent follow-up is needed for more patients with co-expression of PD-L1 and SOCS3. In addition, a combinational therapy with SOCS3 stimulator and PD-L1 blockade may be beneficial for HCC patients and needs to be examined in future studies.

## Figures and Tables

**Table 1:** Association of the expression of CD4 and CD8 with clinicopathologic characteristics of 74 hepatocellular carcinoma patients who underwent surgical treatment

Characteristic	Patients		CD4 Expression			CD8 Expression		
	No.	%	High-expression		p-value	High-expression		p-value
			No.	%		No.	%	
Total	74	100	36	48.6		40	54.1	
**Gender**					0.621			0.283
Female	6	8.1	4	66.7		5	51.5	
Male	68	91.9	32	47.1		35	83.3	
**Age**					0.106			0.448
≤52	40	54.1	20	50.0		20	50.0	
>52	34	45.9	16	47.1		20	58.8	
**Edmondson grading**					0.990			0.613
I-II	35	47.3	17	48.6		20	57.1	
III-IV	39	52.7	19	48.7		20	51.3	
**Vessel invasion**					0.232			0.498
Yes	19	25.7	7	36.8		9	47.4	
No	55	74.3	29	52.7		31	56.4	
**Tumor size**					0.737			0.326
>5cm	24	32.4	11	45.8		11	45.8	
≤5cm	50	67.6	25	50.0		29	58.0	
**Tumor number**					0.945			0.525
1	68	91.9	33	48.5		38	55.9	
2	6	8.1	3	50.0		2	33.3	
**Tumor capsule**					0.153			0.138
Complete	33	44.6	13	39.4		21	63.6	
Incomplete	41	55.4	23	56.1		19	46.3	
**pT categories**					0.288			0.215
pT1	49	66.2	26	53.1		29	59.2	
pT2/pT3	25	33.8	10	40.0		11	44.0	
**Cirrhosis**					0.769			0.808
Positive	66	89.2	33	50.0		36	54.5	
Negative	8	10.8	3	37.5		4	50.0	
**HBsAg**					0.687			0.315
Positive	57	77.0	27	47.4		29	50.9	
Negative	17	23.0	9	52.9		11	64.7	
**TBiL**					0.418			0.987
3.5-20umol/L	61	82.4	31	50.8		33	54.1	
>20umol/L	13	17.6	5	38.5		7	53.8	
**ALT**					0.790			0.121
0-40 U/L	42	56.8	21	50.0		26	61.9	
>40 U/L	32	43.2	15	46.9		14	43.8	
**AFP**					0.247			0.801
<100ug/L	38	51.4	16	42.1		20	52.6	
≥100ug/L	36	48.6	20	55.6		20	55.6	

*p-value<0.05.

**Table 2:** Association of the expression of PD-L1, CD276 and SOCS3 with clinicopathologic characteristics of 74 hepatocellular carcinoma patients who underwent surgical treatment

Characteristic	Patients		PD-L1 Expression			CD276 Expression			SOCS3 Expression		
	No.	%	High-expression		p-value	High-expression		p-value	High-expression		p-value
			No.	%		No.	%		No.	%	
Total	74	100	26	35.1		38	51.4		49	66.2	
**Gender**					0.923			0.178			0.981
Female	6	8.1	2	33.3		1	16.7		4	66.7	
Male	68	91.9	24	35.3		37	54.4		45	66.2	
**Age**					0.644			0.801			0.026^*^
≤52	40	54.1	15	37.5		20	50.0		31	77.5	
>52	34	45.9	11	32.4		18	52.9		18	52.9	
**Edmondson grading**					<0.001^*^			0.021^*^			0.685
I-II	35	47.3	5	14.3		13	37.1		24	68.6	
III-IV	39	52.7	21	53.8		25	64.1		25	64.1	
**Vessel invasion**					0.350			0.897			0.374
Yes	19	25.7	5	26.3		10	52.6		11	57.8	
No	55	74.3	21	38.2		28	50.9		38	69.1	
**Tumor size**					0.822			0.405			0.041^*^
>5cm	24	32.4	8	33.3		14	58.3		12	50.0	
≤5cm	50	67.6	18	36.0		24	48.0		37	74.0	
**Tumor number**					0.214			0.227			0.635
1	68	91.9	22	32.4		33	48.5		44	64.7	
2	6	8.1	4	66.7		5	83.3		5	83.3	
**Tumor capsule**					0.078			0.658			0.360
Complete	33	44.6	8	24.2		16	48.5		20	60.6	
Incomplete	41	55.4	18	43.9		22	53.7		29	70.7	
**pT categories**					0.911			0.288			0.773
pT1	49	66.2	17	34.7		23	46.9		33	67.3	
pT2/pT3	25	33.8	9	36.0		15	60.0		16	64.0	
**Cirrhosis**					0.304			0.228			0.341
Positive	66	89.2	25	37.9		36	54.5		42	63.6	
Negative	8	10.8	1	12.5		2	25.0		7	87.5	
**HBsAg**					0.241			0.881			0.187
Positive	57	77.0	18	31.6		29	50.9		40	70.2	
Negative	17	23.0	8	47.1		9	52.9		9	52.9	
**TBiL**					0.186			0.102			0.800
3.5-20 umol/L	61	82.4	24	39.3		34	55.7		40	65.6	
>20 umol/L	13	17.6	2	15.4		4	30.8		9	69.2	
**ALT**					0.037^*^			0.107			0.555
0-40 U/L	42	56.8	19	45.2		25	59.5		29	69.0	
>40 U/L	32	43.2	7	21.9		13	40.6		20	62.5	
**AFP**					0.103			0.481			0.936
<100 ug/L	38	51.4	10	26.3		18	47.4		25	65.8	
≥100 ug/L	36	48.6	16	44.4		20	55.6		24	66.7	
**CD4 expression**					0.422			0.481			0.059
Low	38	51.4	15	39.5		18	47.4		29	76.3	
High	36	48.6	11	30.6		20	55.6		20	55.6	
**CD8 expression**					0.644			0.801			0.083
Low	34	45.9	11	32.4		18	52.9		19	55.9	
High	40	54.1	15	37.5		20	50.0		30	75.0	
**CD4/CD8 expression**					0.966			0.684			0.683
Low	23	31.1	8	34.8		11	47.8		16	69.6	
High	51	68.9	18	24.3		27	52.9		33	64.7	

*p-value<0.05.

**Table 3:** Univariate cox regression analyses of pathologic features and molecular markers for the overall survival of 74 hepatocellular carcinoma patients who underwent surgical treatment

Variables	Univariate		
	Hazard Ratio	95%CI	p-value
Gender (female vs. male)	0.336	0.046-2.466	0.284
Age (<52y vs. ≥52y)	0.606	0.301-1.219	0.160
Edmondson grading (I-II vs. III-IV)	3.029	1.398-6.563	0.005^*^
Vessel invasion (positive vs. negative)	1.296	0.613-2.739	0.497
Tumor size (≤5cm vs. >5cm)	1.812	0.900-3.648	0.096
Tumor number (1 vs. 2)	3.705	1.501-9.144	0.004^*^
Tumor capsule (complete vs. incomplete)	1.908	0.919-3.961	0.083
pT categories (pT1 vs. pT2/pT3)	2.139	1.066-4.289	0.032^*^
Cirrhosis (positive vs. negative)	2.046	0.489-8.568	0.327
HBsAg (positive vs. negative)	1.142	0.513-2.542	0.745
TBiL (3.5-20umol/L vs. >20umol/L)	1.213	0.467-3.151	0.691
ALT (0-40U/L vs. >40U/L)	1.220	0.609-2.445	0.574
AFP (<100ug/L vs. ≥100ug/L)	1.365	0.681-2.735	0.380
CD4/CD8 expression (high vs. low)	0.559	0.276-1.135	0.107
PD-L1 expression (high vs. low)	2.754	1.373-5.525	0.004^*^
CD276 expression (high vs. low)	1.633	0.806-3.309	0.174
SOCS3 expression (high vs. low)	0.435	0.217-0.872	0.019^*^

*p-value<0.05.

**Table 4:** Multivariate cox regression analyses of pathologic features and molecular markers for the overall survival of 74 hepatocellular carcinoma patients who underwent surgical treatment

Variables	Multivariate		
	Hazard Ratio	95%CI	p-value
pT categories (pT1 vs. pT2/pT3)	2.122	1.108-4.379	0.045^*^
Edmondson grading (I-II vs. III-IV)	1.718	0.742-3.980	0.207
Tumor capsule (complete vs. incomplete)	1.153	0.501-2.652	0.737
CD4/CD8 expression (high vs. low)	0.455	0.214-0.966	0.040^*^
PD-L1 expression (high vs. low)	5.275	2.506-13.082	<0.001^*^
SOCS3 expression (high vs. low)	0.210	0.093-0.475	<0.001^*^

*p-value<0.05.

## Abbreviations

HCC: hepatocellular carcinoma
HBV: hepatitis B virus
PD-1: programmed cell death protein 1
PD-L1: programmed cell death 1 ligand 1
PD-L2: programmed cell death protein ligand 2
TME: tumor microenvironment
CTL: cytotoxic T lymphocyte
Tregs: regulatory T cells
TILs: tumor-infiltrating lymphocytes
TCR: T cell receptor
SOCS3: suppressor of cytokine signaling 3
GEO: gene expression omnibus
FFPE: formalin-fixed: paraffin-embedded tumors
AJCC: American Joint Committee on Cancer
IHC: immunohistochemistry
OS: overall survival
